# Inequalities in access to minimally invasive general surgery: a comprehensive nationwide analysis across 20 years

**DOI:** 10.1007/s00464-020-08123-0

**Published:** 2020-11-18

**Authors:** Marcel André Schneider, Daniel Gero, Matteo Müller, Karoline Horisberger, Andreas Rickenbacher, Matthias Turina

**Affiliations:** Department of Surgery, University Hospital Zurich, Raemistrasse 100, 8091 Zurich, Switzerland

**Keywords:** Minimally invasive surgery, Laparoscopy, Robotic surgery, Outcomes, Appendectomy, Cholecystectomy, Colectomy, Rectal resection, Gastrectomy

## Abstract

**Background:**

Minimally invasive surgery (MIS) has profoundly changed standards of care and lowered perioperative morbidity, but its temporal implementation and factors favoring MIS access remain elusive. We aimed to comprehensibly investigate MIS adoption across different surgical procedures over 20 years, identify predictors for MIS amenability and compare propensity score-matched outcomes among MIS and open surgery.

**Methods:**

Nationwide retrospective analysis of all hospitalizations in Switzerland between 1998 and 2017. Appendectomies (*n* = 186,929), cholecystectomies (*n* = 57,788), oncological right (*n* = 9138) and left hemicolectomies (*n* = 21,580), rectal resections (*n* = 13,989) and gastrectomies for carcinoma (*n* = 6606) were included. Endpoints were assessment of temporal MIS implementation, identification of predictors for MIS access and comparison of propensity score-matched outcomes among MIS and open surgery.

**Results:**

The rates of MIS increased for all procedures during the study period (*p* ≤ 0.001). While half of all appendectomies were performed laparoscopically by 2005, minimally invasive oncological colorectal resections reached 50% only by 2016. Multivariate analyses identified older age (*p* ≤ 0.02, except gastrectomy), higher comorbidities (*p* ≤ 0.001, except rectal resections), lack of private insurance (*p* ≤ 0.01) as well as rural residence (*p* ≤ 0.01) with impaired access to MIS. Rural residence correlated with low income regions (*p* ≤ 0.001), which themselves were associated with decreased MIS access. Geographical mapping confirmed strong disparities for rural and low-income areas in MIS access. Matched outcome analyses revealed benefits of MIS for length of stay, decreased surgical site infection rates for MIS appendectomies and cholecystectomies and higher mortality for open cholecystectomies. No consistent morbidity or mortality benefit for MIS compared to open colorectal resections was observed.

**Conclusion:**

Unequal access to MIS exists in disfavor of older and more comorbid patients and those lacking private insurance, living in rural areas, and having lower income. Efforts should be made to ensure equal MIS access regardless of socioeconomic or geographical factors.

**Electronic supplementary material:**

The online version of this article (10.1007/s00464-020-08123-0) contains supplementary material, which is available to authorized users.

Implementation of minimally invasive surgery (MIS) has marked a major step forward in the surgical treatment of various diseases over the last decades [1]. Initially used in less demanding operations such as appendectomies or cholecystectomies, MIS has swiftly been adopted to increasingly complex procedures such as oncological colectomies [2], pulmonary lobectomies [3] or gastrectomies [4], providing benefits with respect to return of bowel function, pain, cosmetic results, length of hospital stay (LOS), rates of complications [5] and cost-effectiveness [6].

Regarding oncological outcomes, multiple randomized trials [7–11] and observational studies have shown that laparoscopic approaches for resections of colorectal cancer (CRC) provide equal oncological quality and long-term survival as conventional open surgery (OS). Similar level 1 evidence of equal outcomes were reported for gastrectomies [12–15], lobectomies [16], distal pancreatectomies [17] and resection of colorectal liver metastases [18]. However, recent studies have also shown shortcomings of MIS in operations such as hysterectomies [19, 20] or pancreaticoduodenectomies [21] with regard to complications and long-term oncological results, although multiple earlier meta-analyses have suggested similar outcomes [22].

While the use of MIS increases steadily [23], United States (US)-based observational studies objectified that major hurdles and inequalities in access to MIS remain. Disparities in choice of surgical access exist based on patient related factors such as ethnicity [24] or insurance status [25, 26] as well as patient-independent factors such as the treating hospital, experience of the surgeon and geographical residential area [27]. In contrast, comprehensive analyses of factors influencing the choice for or against MIS in Europe are currently missing. Furthermore, it remains elusive at which pace MIS approaches were implemented following the published evidence of non-inferior outcomes. While multiple studies reporting on institutional developments of MIS techniques exist, long-term nationwide analyses remain scarce.

Using a national surgical quality control database, we have previously reported that patients in Europe with private insurance have a higher likelihood to receive a MIS colorectal resection [28]. Here, we aimed to investigate the nationwide use and implementation of MIS over a 20-year period across different general surgical procedures. We hypothesized that individual patient access to MIS techniques varies depending on demographic, socioeconomic and geographical factors.

## Methods

### Study design and data source

The current study is a retrospective observational, nationwide analysis of patients undergoing appendectomies, cholecystectomies and oncological colorectal and gastric resections over 20 years. The Swiss federal statistical office’s (BFS, Neuchatel, Switzerland) databases covering the mandatory, nationwide reporting of all stationary hospitalizations (≥ 24 h) in Swiss hospitals starting from 1998 to 2017 were queried. These databases contain anonymized patient-level data including the main diagnosis responsible for hospitalization and up to 49 secondary diagnoses for comorbidities and complications coded via *International Classification of Diseases* (ICD-10 German modification) definitions. Procedures are coded by national Swiss surgical classification codes (CHOP), issued annually by the BFS classifying all medical interventions [29].

Data on age (5-year categories), gender, nationality, insurance status are provided as categorial variables. The databases provide place of residence of patients with concomitant anonymity of single cases in 706 subdistrict geographical clusters (MedStat regions) containing one or several official political municipalities [30], with exact information on place of residence (e.g. ZIP codes), location, caseload or case-mix of the treating hospital not available due to anonymization. Each Swiss municipality is classified as urban, suburban or rural [31] and MedStat regions and the included cases were classified accordingly by merging to corresponding municipalities (majority vote in case of differences). Similarly, mean taxable income per municipality in 2015 was queried of the Swiss federal tax administration [32] and averaged on overlapping MedStat regions. For population-adjusted rates of operations, we obtained the total number of inhabitants in Switzerland per year by the BFS [33].

### Data processing

The databases were searched for cases of hospitalisations based on the respective main diagnoses acute appendicitis, cholecystitis, colorectal and gastric carcinoma according to the corresponding annual ICD-10 definitions and consequently filtered for the surgical procedures of interest by respective year-matched CHOP codes (Supplementary Tables 1, 2). All codes were specified at study start by consensus of 3 investigators (MS, DG, MT). Minimally invasive or conventional open procedures were distinguished based on codes specifying the surgical approach or codes indicating a laparoscopic access or use of robotic surgical system. Cases were grouped as open, laparoscopic and robotic to assess trends over time. For further analyses, laparoscopic and robotic approaches were combined into MIS cases and compared to OS. For oncological resections, cases were restricted to patients undergoing elective procedures. Readmissions and cases, in which the main reason for surgery was due to a complication, were excluded. Comorbidities & complications were assessed via 49 reported ICD side codes (Supplementary Table 3) and used to assess the extent of comorbidities via the modified version [34] of the Elixhauser score [35].

**Table 1:** Baseline patient demographic data

	Appendectomy (*n* = 186,929)			Cholecystectomy (*n* = 57,788)			Right hemicolectomy (*n* = 9138)		
	OS	MIS	*p*-value	OS	MIS	*p*-value	OS	MIS	*p*-value
	(*n* = 58,964)	(*n* = 127,965)		(*n* = 6831)	(*n* = 50,957)		(*n* = 7362)	(*n* = 1776)	
Gender									
Male	36,696 (62.2%)	64,710 (50.6%)	< 0.001	3910 (57.2%)	24,165 (47.4%)	< 0.001	3738 (50.8%)	894 (50.3%)	0.751
Female	22,268 (37.8%)	63,255 (49.4%)		2921 (42.8%)	26,792 (52.6%)		3624 (49.2%)	882 (49.7%)	
Age (years)									
00–19	26,155 (44.4%)	35,812 (28.0%)	< 0.001	15 (0.2%)	308 (0.6%)	< 0.001	3 (0.0%)	1 (0.1%)	0.173
20–39	16,086 (27.3%)	50,056 (39.1%)		300 (4.4%)	7675 (15.1%)		102 (1.4%)	19 (1.1%)	
40–59	10,459 (17.7%)	28,765 (22.5%)		1358 (19.9%)	18,163 (35.6%)		953 (12.9%)	252 (14.2%)	
60–79	5155 (8.7%)	11,707 (9.1%)		3440 (50.4%)	19,903 (39.1%)		4036 (54.8%)	1001 (56.4%)	
80–99	1109 (1.9%)	1625 (1.3%)		1718 (25.2%)	4908 (9.6%)		2268 (30.8%)	503 (28.3%)	
Nationality									
Swiss	46,732 (79.3%)	96,283 (75.2%)	< 0.001	5828 (85.3%)	40,921 (80.3%)	< 0.001	6490 (88.2%)	1513 (85.2%)	< 0.001
Foreign	10,011 (17.0%)	30,519 (23.8%)		881 (12.9%)	9322 (18.3%)		816 (11.1%)	262 (14.8%)	
Not available	2221 (3.8%)	1163 (0.9%)		122 (1.8%)	714 (1.4%)		56 (0.8%)	1 (0.1%)	
Area of residence									
Urban City	12,170 (20.6%)	33,004 (25.8%)	< 0.001	1530 (22.4%)	12,200 (23.9%)	< 0.001	1826 (24.8%)	492 (27.7%)	< 0.001
Smaller towns and suburbs	21,910 (37.2%)	53,651 (41.9%)		2619 (38.3%)	21,730 (42.6%)		3086 (41.9%)	841 (47.4%)	
Rural area	19,238 (32.6%)	34,656 (27.1%)		2235 (32.7%)	13,839 (27.2%)		1995 (27.1%)	408 (23.0%)	
Abroad	5646 (9.6%)	6654 (5.2%)		447 (6.5%)	3188 (6.3%)		455 (6.2%)	35 (2.0%)	
Taxable income per region									
0–19 Percentile	12,849 (21.8%)	21,947 (17.2%)	< 0.001	1464 (21.4%)	9311 (18.3%)	< 0.001	1472 (20.0%)	300 (16.9%)	< 0.001
20–39 Percentile	11,439 (19.4%)	23,492 (18.4%)		1399 (20.5%)	9484 (18.6%)		1416 (19.2%)	290 (16.3%)	
40–59 Percentile	10,204 (17.3%)	24,649 (19.3%)		1218 (17.8%)	9535 (18.7%)		1315 (17.9%)	336 (18.9%)	
60–79 Percentile	9547 (16.2%)	25,464 (19.9%)		1104 (16.2%)	9773 (19.2%)		1363 (18.5%)	384 (21.6%)	
80–100 Percentile	9279 (15.7%)	25,759 (20.1%)		1199 (17.6%)	9666 (19.0%)		1341 (18.2%)	431 (24.3%)	
Missing	5646 (9.6%)	6654 (5.2%)		447 (6.5%)	3188 (6.3%)		455 (6.2%)	35 (2.0%)	

	Left hemicolectomy (*n* = 21,580)			Rectal resection (*n* = 13,989)			Gastrectomy (*n* = 6606)		
	OS	MIS	*p*-value	OS	MIS	*p*-value	OS	MIS	*p*-value
	(*n* = 15,836)	(*n* = 5744)		(*n* = 10,241)	(*n* = 3748)		(*n* = 6086)	(*n* = 520)	
Gender									
Male	9383 (59.3%)	3372 (58.7%)	0.471	6390 (62.4%)	2317 (61.8%)	0.542	3660 (60.1%)	304 (58.5%)	0.456
Female	6453 (40.7%)	2372 (41.3%)		3851 (37.6%)	1431 (38.2%)		2426 (39.9%)	216 (41.5%)	
Age (years)									
00–19	2 (0.0%)	4 (0.1%)	< 0.001	0 (0%)	0 (0%)	< 0.001	1 (0.0%)	0 (0%)	0.630
20–39	205 (1.3%)	90 (1.6%)		152 (1.5%)	85 (2.3%)		175 (2.9%)	18 (3.5%)	
40–59	3074 (19.4%)	1303 (22.7%)		2335 (22.8%)	985 (26.3%)		1470 (24.2%)	127 (24.4%)	
60–79	9428 (59.5%)	3417 (59.5%)		6094 (59.5%)	2141 (57.1%)		3377 (55.5%)	297 (57.1%)	
80–99	3127 (19.7%)	930 (16.2%)		1660 (16.2%)	537 (14.3%)		1063 (17.5%)	78 (15.0%)	
Nationality									
Swiss	13,594 (85.8%)	4826 (84.0%)	< 0.001	8811 (86.0%)	3131 (83.5%)	< 0.001	4626 (76.0%)	400 (76.9%)	0.917
Foreign	2037 (12.9%)	905 (15.8%)		1334 (13.0%)	614 (16.4%)		1370 (22.5%)	113 (21.7%)	
Not available	205 (1.3%)	13 (0.2%)		96 (0.9%)	3 (0.1%)		90 (1.5%)	7 (1.3%)	
Area of residence									
Urban City	4003 (25.3%)	1542 (26.8%)	< 0.001	2578 (25.2%)	1007 (26.9%)	< 0.001	1485 (24.4%)	166 (31.9%)	< 0.001
Smaller towns and suburbs	6416 (40.5%)	2632 (45.8%)		4005 (39.1%)	1639 (43.7%)		2479 (40.7%)	205 (39.4%)	
Rural area	4349 (27.5%)	1399 (24.4%)		2981 (29.1%)	1003 (26.8%)		1686 (27.7%)	130 (25.0%)	
Abroad	1068 (6.7%)	171 (3.0%)		677 (6.6%)	99 (2.6%)		436 (7.2%)	19 (3.7%)	
Taxable income per region									
0–19 Percentile	3165 (20.0%)	960 (16.7%)	< 0.001	1950 (19.0%)	673 (18.0%)	< 0.001	1141 (18.7%)	77 (14.8%)	0.011
20–39 Percentile	2936 (18.5%)	1016 (17.7%)		2089 (20.4%)	688 (18.4%)		1134 (18.6%)	86 (16.5%)	
40–59 Percentile	2986 (18.9%)	1083 (18.9%)		1959 (19.1%)	668 (17.8%)		1111 (18.3%)	120 (23.1%)	
60–79 Percentile	2675 (16.9%)	1229 (21.4%)		1840 (18.0%)	926 (24.7%)		1138 (18.7%)	113 (21.7%)	
80–100 Percentile	3006 (19.0%)	1285 (22.4%)		1726 (16.9%)	694 (18.5%)		1126 (18.5%)	105 (20.2%)	
Missing	1068 (6.7%)	171 (3.0%)		677 (6.6%)	99 (2.6%)		436 (7.2%)	19 (3.7%)	

*OS* open surgery, *MIS* minimally invasive surgery

**Table 2:** Baseline surgical and hospitalisation data

	Appendectomy (*n* = 186,929)			Cholecystectomy (*n* = 57,788)			Right hemicolectomy (*n* = 9138)		
	OS	MIS	*p*-value	OS	MIS	*p*-value	OS	MIS	*p*-value
	(*n* = 58,964)	(*n* = 127,965)		(*n* = 6831)	(*n* = 50,957)		(*n* = 7362)	(*n* = 1776)	
Surgical technique									
Open surgery	58,964 (100%)	0 (0%)	< 0.001	6831 (100%)	0 (0%)	< 0.001	7362 (100%)	0 (0%)	< 0.001
Laparoscopic surgery	0 (0%)	127,965 (100%)		0 (0%)	50,926 (99.9%)		0 (0%)	1741 (98.0%)	
Robotic surgery	0 (0%)	0 (0%)		0 (0%)	31 (0.1%)		0 (0%)	35 (2.0%)	
Year of operation									
1998–2002	23,435 (39.7%)	10,826 (8.5%)	< 0.001	1985 (29.1%)	8189 (16.1%)	< 0.001	1525 (20.7%)	37 (2.1%)	< 0.001
2003–2007	21,854 (37.1%)	24,633 (19.2%)		1827 (26.7%)	11,156 (21.9%)		1894 (25.7%)	223 (12.6%)	
2008–2012	10,614 (18.0%)	41,338 (32.3%)		1542 (22.6%)	14,499 (28.5%)		2053 (27.9%)	526 (29.6%)	
2013–2017	3061 (5.2%)	51,168 (40.0%)		1477 (21.6%)	17,113 (33.6%)		1890 (25.7%)	990 (55.7%)	
Insurance status									
Statutory	50,364 (85.4%)	105,328 (82.3%)	< 0.001	5117 (74.9%)	36,811 (72.2%)	< 0.001	4874 (66.2%)	1089 (61.3%)	< 0.001
Private	8435 (14.3%)	22,415 (17.5%)		1702 (24.9%)	14,083 (27.6%)		2482 (33.7%)	687 (38.7%)	
Not available	165 (0.3%)	222 (0.2%)		12 (0.2%)	63 (0.1%)		6 (0.1%)	0 (0%)	
Length of hospital stay (days)									
Mean (SD)	4.71 (5.43)	3.60 (3.21)	< 0.001	12.3 (9.56)	5.78 (8.18)	< 0.001	15.9 (10.4)	14.1 (12.1)	< 0.001
Median [Q1, Q3]	4.00 [2.00, 5.00]	3.00 [2.00, 4.00]	< 0.001	10.0 [7.00, 15.0]	4.00 [3.00, 7.00]	< 0.001	13.0 [10.0, 19.0]	10.0 [7.00, 17.0]	< 0.001
Missing	0 (0%)	2 (0.0%)		2 (0.0%)	0 (0%)		0 (0%)	0 (0%)	
Comorbidities (Elixhauser Score)									
Mean (SD)	0.243 (2.21)	0.283 (2.40)	< 0.001	2.62 (6.41)	0.806 (4.20)	< 0.001	11.7 (7.42)	12.6 (8.23)	< 0.001
Median [Q1, Q3]	0.00 [0.00, 0.00]	0.00 [0.00, 0.00]	< 0.001	0.00 [0.00, 4.00]	0.00 [0.00, 0.00]	< 0.001	7.00 [7.00, 14.0]	10.0 [7.00, 16.0]	0.007
In-hospital mortality									
No	58,878 (99.9%)	127,909 (100.0%)	< 0.001	6689 (97.9%)	50,817 (99.7%)	< 0.001	7092 (96.3%)	1721 (96.9%)	0.254
Yes	86 (0.1%)	56 (0.0%)		142 (2.1%)	140 (0.3%)		270 (3.7%)	55 (3.1%)	

	Left Hemicolectomy (*n* = 21,580)			Rectal Resection (*n* = 13,989)			Gastrectomy (*n* = 6606)		
	OS	MIS	*p*-value	OS	MIS	*p*-value	OS	MIS	*p*-value
	(*n* = 15,836)	(*n* = 5744)		(*n* = 10,241)	(n = 3748)		(*n* = 6086)	(*n* = 520)	
Surgical technique									
Open surgery	15,836 (100%)	0 (0%)	< 0.001	10,241 (100%)	0 (0%)	< 0.001	6086 (100%)	0 (0%)	< 0.001
Laparoscopic surgery	0 (0%)	5683 (98.9%)		0 (0%)	3550 (94.7%)		0 (0%)	485 (93.3%)	
Robotic surgery	0 (0%)	61 (1.1%)		0 (0%)	198 (5.3%)		0 (0%)	35 (6.7%)	
Year of operation									
1998–2002	3831 (24.2%)	265 (4.6%)	< 0.001	2371 (23.2%)	119 (3.2%)	< 0.001	1499 (24.6%)	43 (8.3%)	< 0.001
2003–2007	4559 (28.8%)	815 (14.2%)		2901 (28.3%)	439 (11.7%)		1609 (26.4%)	83 (16.0%)	
2008–2012	3981 (25.1%)	2080 (36.2%)		2626 (25.6%)	1177 (31.4%)		1542 (25.3%)	168 (32.3%)	
2013–2017	3465 (21.9%)	2584 (45.0%)		2343 (22.9%)	2013 (53.7%)		1436 (23.6%)	226 (43.5%)	
Insurance status									
Statutory	10,735 (67.8%)	3717 (64.7%)	< 0.001	7014 (68.5%)	2505 (66.8%)	0.008	4570 (75.1%)	355 (68.3%)	0.003
Private	5077 (32.1%)	2026 (35.3%)		3214 (31.4%)	1243 (33.2%)		1510 (24.8%)	165 (31.7%)	
Not available	24 (0.2%)	1 (0.0%)		13 (0.1%)	0 (0%)		6 (0.1%)	0 (0%)	
Length of hospital stay (days)									
Mean (SD)	16.3 (10.7)	13.0 (11.5)	< 0.001	18.6 (13.1)	16.2 (13.4)	< 0.001	21.1 (16.0)	18.5 (18.6)	0.002
Median [Q1, Q3]	14.0 [10.0, 19.0]	9.00 [7.00, 15.0]	< 0.001	15.0 [12.0, 21.0]	12.0 [9.00, 19.0]	< 0.001	17.0 [13.0, 24.0]	14.0 [9.00, 21.0]	< 0.001
Missing	1 (0.0%)	2 (0.0%)		2 (0.0%)	1 (0.0%)		0 (0%)	0 (0%)	
Comorbidities (Elixhauser Score)									
Mean (SD)	11.3 (7.16)	11.7 (7.42)	< 0.001	10.8 (6.83)	12.3 (7.63)	< 0.001	11.8 (7.36)	12.4 (8.21)	0.106
Median [Q1, Q3]	7.00 [7.00, 14.0]	9.00 [7.00, 14.0]	0.001	7.00 [7.00, 14.0]	10.0 [7.00, 16.0]	< 0.001	7.00 [7.00, 14.0]	9.00 [7.00, 16.0]	0.704
In-hospital mortality									
No	15,349 (96.9%)	5622 (97.9%)	< 0.001	9998 (97.6%)	3682 (98.2%)	0.032	5818 (95.6%)	508 (97.7%)	0.023
Yes	487 (3.1%)	122 (2.1%)		243 (2.4%)	66 (1.8%)		268 (4.4%)	12 (2.3%)	

*OS* open surgery, *MIS* minimally invasive surgery

### Outcomes of interest

Primary endpoint was the assessment of implementation of minimally invasive approaches (laparoscopic and robotic) over a 20-year period and identification of predictors for MIS approach. Secondary endpoint was comparison of short-term surgical outcomes of matched OS and MIS cases, including LOS, complications, and in-hospital mortality.

### Ethics approval and written consent

As all information in the database are provided completely anonymized by the BFS, no institutional ethics approval or individual patient written consent was required according to the current Swiss Human Research Act.

### Statistical analysis

Statistical significance was defined as *p* < 0.05. Continuous data are given as mean ± standard deviation (SD) or median ± interquartile range (IQR) as appropriate and categorical as number (*n*) and percentage (%). Students *t*-test, Wilcoxon’s rank sum test or Fisher’s exact test was used to compare means, medians and proportions or odds among groups. Mann–Kendall Trend Test served to test changes in frequency over time. Correlation between numerical variables was assessed with Pearson’s correlation coefficient. Logistic regression with binary outcome “open” vs. “minimally invasive” procedure served to identify factors influencing the choice of the surgical approach and respective odds with 95% confidence intervals (CI). Propensity score matching was performed using the nearest neighbor method (logistic regression distance, caliper: 0.1) based on year of the operation and identified factors influencing the choice of surgical approach. A 1:1 ratio for interventions with high MIS frequency was used, while allowing up to 5:1 matching for procedures with low MIS frequencies with possible discarding of extreme cases in both groups. Geographical mapping was based on respective MedStat regions of patients by displaying frequencies of MIS approached on publicly available shapefile maps. *R* version 3.5.1 was used for all database processing, statistical analyses, and graphical representations.

## Results

### Identification of surgical procedures and developments over time

**Fig. 1:**
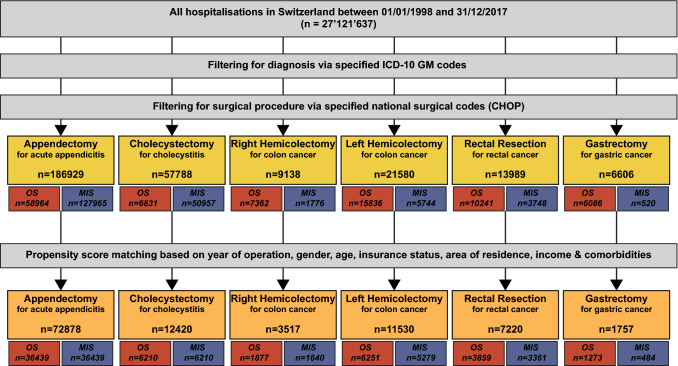
Flowchart of patient identification per surgical procedure, depicting the complete dataset, total numbers of identified patients per surgical procedure with stratification of OS and MIS procedures and respective numbers after propensity score matching. *OS* open surgery, *MIS* minimally invasive surgery

The combined databases comprised 27,121,637 hospitalizations from 1st of January 1998 to 31st of December 2017. Based on respective yearly ICD-10 and surgical codes, 186,929 appendectomies, 57,788 cholecystectomies, 9138 oncological right and 21,580 left hemicolectomies, 13,989 rectal resections and 6606 gastrectomies for carcinoma were identified (Fig. 1). Table 1 summarizes the demographic baseline data of patients per procedure stratified according to OS or MIS, while Table 2 provides data on hospitalization parameters.

**Fig. 2:**
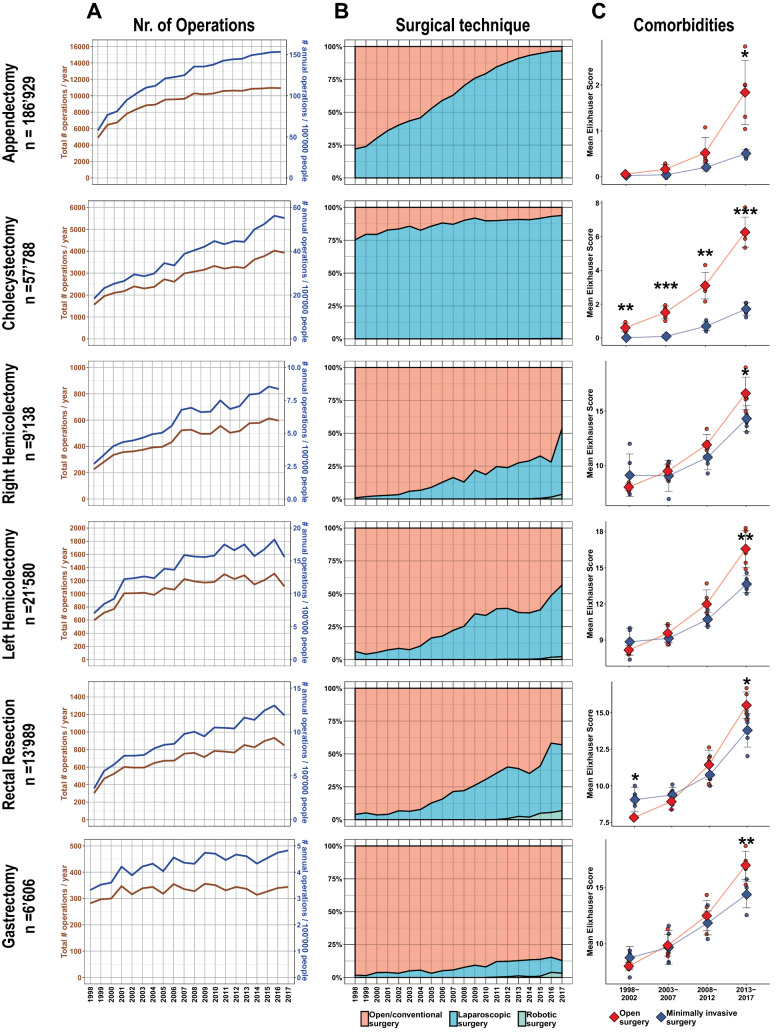
Number of operations, implementation of minimally invasive procedures and comorbidities. **A** Total (brown) and population adjusted (blue) numbers of procedures performed per year from 1998 to 2017. **B** Representation of percent changes of open (red), laparoscopic (turquois) and robotic (green) surgeries over the 20-year period. **C** Comorbidities as assessed with Elixhauser score for open (dark red) and minimally invasive (blue) surgeries per procedure in 5-year strata. Single points depict yearly means, diamond & error bars show mean and SD of 5 years. *p < 0.05, **p < 0.01, ***p ≤ 0.001

The number of total procedures as well as population-adjusted procedure rates per year increased during 2008–2017 for appendectomies, cholecystectomies, right hemicolectomies, and rectal resections (*p* < 0.01), while those for left hemicolectomies and gastrectomies remained stable (Fig. 2A). We found different proportions of MIS procedures over the whole 20-year period, ranging from 7.8% for gastrectomies, 19.4% to 26.7% for colorectal resections, 68.5% for appendectomies and 88.1% for cholecystectomies. The rates of MIS increased across all procedures during the study period (*p* < 0.001), with incremental amounts of robotic procedures observed for all 4 oncological operations. Cholecystectomy was already routinely (> 75%) performed laparoscopically in 1998. While half of all appendectomies were performed laparoscopically by 2005, minimally invasive oncological colorectal resections reached 50% by 2016 and stayed below 20% for gastrectomies in 2017 (Fig. 2B). Next, Elixhauser scores were calculated to assess comorbidities of patients undergoing OS and MIS and compared over groups of 5-year intervals. The degrees of comorbidities increased across all procedures studied, with OS being preferably used in patients with higher Elixhauser scores in all 6 procedures. For cholecystectomies, rates of OS were significantly higher during the entire 20 years in patients with increased comorbidities (Fig. 2C).

### Predictors of MIS access

**Fig. 3:**
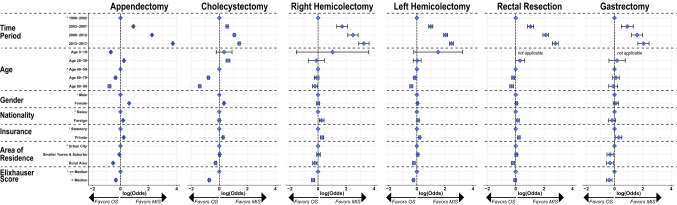
Odds of factors predicting minimally invasive access per surgical procedure. Graphical representation of logistic regression results regarding choice of MIS vs. OS for all 6 procedures. Blue diamonds indicate logistic odds compared to reference levels with error bars showing 95% CI. *Reference levels. *OS* open surgery, *MIS* minimally invasive surgery

Using logistic regression, we explored socioeconomic and demographic factors influencing the choice of surgical access (Fig. 3). The likelihood of receiving a MIS procedure increased greatly over time for all procedures. Patients were 9 × more likely to have a MIS appendectomy during 2008–2012 and 41 × more likely during 2013–2017 compared to 1998–2002. Similarly, odds increased 26 × for right hemicolectomy, 11 × for left hemicolectomy, 16 × for rectal resection and 7 × for gastrectomy between the first and last 5-year strata. For cholecystectomy, the increase was only 4 ×, as already a large proportion of procedures was conducted MIS by the beginning of the study period. Older age predisposed to open surgical procedures across all interventions studied. Indeed, patients in the highest group aged 80–99 years were half as likely to receive a MIS appendectomy and had 4 × lower odds for MIS cholecystectomy (both *p* ≤ 0.001). Similarly, odds to receive MIS for the oldest group were decreased by 23% for right hemicolectomy (*p* = 0.02), 48% for left hemicolectomy and 32% for rectal resection (both *p* ≤ 0.001). Similarly, higher comorbidities predisposed to OS with 28% to > 50% lower odds of receiving MIS (all *p* ≤ 0.001), except for patients with rectal resections (*p* = 0.32). For non-oncological operations, female patients were considerably more likely to receive a MIS procedure than their male counterparts. However, this phenomenon was not observed for oncological operations. Patients without Swiss citizenship were more likely to have a MIS appendectomy (*p* ≤ 0.001), right hemicolectomy (*p* = 0.003) and rectal resection (*p* = 0.029), otherwise no clear influence of patients’ nationality was observed. However, patients with private insurance were 22–34% more likely to undergo MIS procedures compared to patients with statutory insurance only (all *p* ≤ 0.001). Patients living in rural areas had impaired access to MIS for all 6 procedures with a 17% to 40% decreased likelihood compared to patients living in urban and suburban areas.

**Fig. 4:**
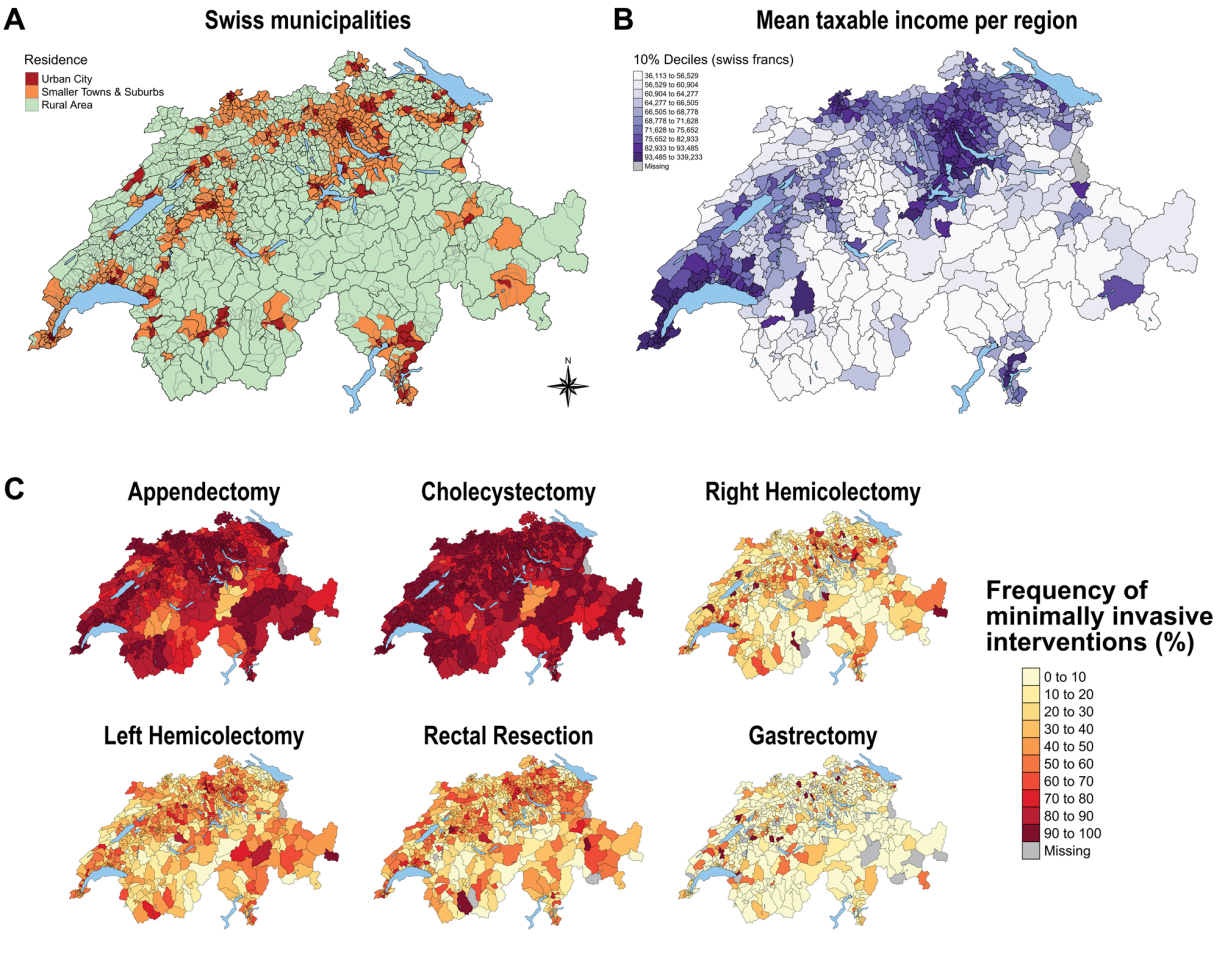
Geographical mapping of MedStat regions based on urban–rural classification of municipalities, income and frequency of minimally invasive interventions. **A** Map of Switzerland showing MedStat regions (black boarders) with underlying municipalities (grey boarders) colored according to classification into urban (red), suburban (orange) and rural (green). **B** Map of Switzerland depicting mean taxable income per MedStat region with color scale of 10%-deciles. **C** Maps of Switzerland depicting frequencies of minimally invasive interventions for all 6 procedures from 2008 to 2017. Note the visual correlation of areas with higher frequencies with urban and high-income areas and vice versa

Next, we attempted to understand the underlying reason for the consistent impaired MIS access in rural patients. Speculating that differences in prosperity and income of regions contribute to the observed differences, we compared mean taxable incomes per regions of different residential areas. Indeed, 48% of rural areas, but only 13% and 11% of suburban and urban areas respectively, grouped in the lowest quintile of incomes. In contrast, the middle quantile of incomes was made up of 15% of rural, 13% suburban and 31% of urban areas, while the top quintile quantile consisted of 8% rural, 22% suburban and 28% of urban areas (*p* ≤ 0.001). Geographical mapping according to urban–rural stratification of regions compared with mapping of mean taxable income per region visually confirmed correlation of urban/suburban areas with higher income across most areas of the country (Fig. 4A, B). Additionally, we found that income itself correlated with frequency of MIS in regions (*R* 0.11–0.13, *p* ≤ 0.001) for appendectomies, cholecystectomies and colectomies, but not for rectal resection and gastrectomies. Furthermore, for all 6 operations, higher mean income per region correlated strongly with increased frequencies of privately insured patients undergoing the procedure (*R* 0.22–0.61, all *p* ≤ 0.001) and all urban and suburban areas had higher frequencies of privately insured patients than rural ones (all *p* ≤ 0.001).

### Geographical differences of MIS implementation

Based on the observed differences among urban and rural regions in MIS access, frequency of MIS per region was calculated from 2008 to 2017 and consequent geographical mapping performed. Compared with maps displaying urban–rural stratification and mean income per regions, we indeed found strong visual correlation of decreased implementation of MIS procedures in rural and low-income areas (Fig. 4C).

### Short-term outcomes of MIS compared to OS

Next, we aimed to identify hospitalisation-related differences among patients undergoing OS vs. MIS. To reliably identify differences owed due to the surgical approach only and not influenced by biasing factors, we propensity score-matched patients undergoing OS or MIS based on the year of operation and identified factors influencing the choice of surgical technique including age, insurance status, comorbidities, urban or rural area and income of region. Matching resulted in groups with attenuated baseline demographic and hospitalization parameters compared to unmatched patients (Supplementary Tables 4, 5) for subsequent analyses of outcomes and complications.

**Fig. 5:**
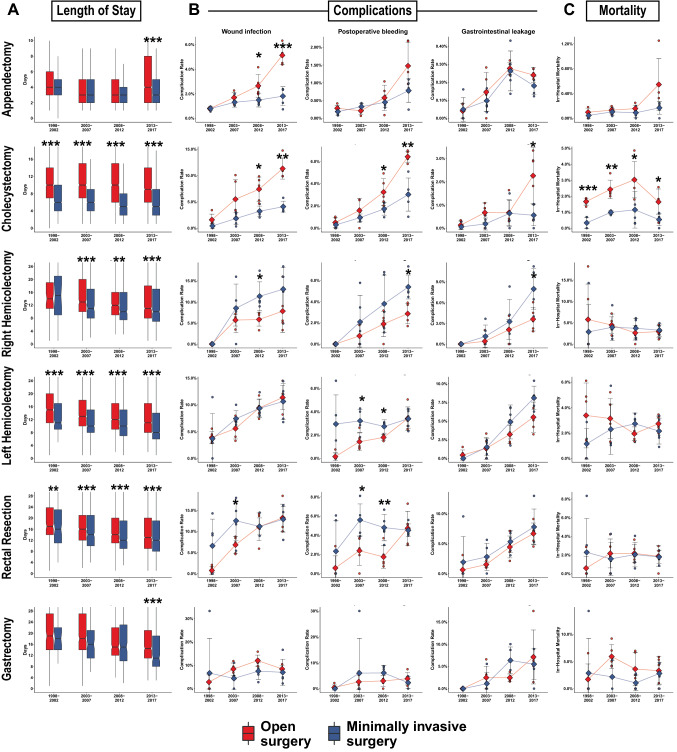
Surgical outcomes of propensity score matched patients. **A** Boxplots showing length of hospital stay of patients operated open (darkred) and minimally invasive (blue) over 5-year strata for all 6 procedures. Rates of selected complication (**B**) and in-hospital mortality (**C**) per intervention for open and minimally invasive procedures. Single points depict yearly means, diamond and error bars show mean and SD of 5 years. *p < 0.05, **p < 0.01, ***p ≤ 0.001

LOS was consistently shorter for all MIS compared to open procedures. With a general short LOS, the observed differences for appendectomies were marginal. In contrast, the effect was most pronounced for patients undergoing cholecystectomy with median LOS of 9–10 days in OS twice the 3–4 days for MIS across the entire 20 years. Patients undergoing MIS hemicolectomies and rectal resections benefitted of a 1–3 days shorter LOS, while MIS gastrectomies reported shortened LOS only in the last period examined (Fig. 5A).

Wound infections were more common in patients undergoing open appendectomy and cholecystectomy, while no clear benefit in this regard was observed for colorectal and gastric resections. Postoperative bleeding was reported more frequently after open cholecystectomy, while inconsistent higher rates were found for MIS colorectal resections. No steady difference was observed for gastrointestinal leakage between MIS and OS for any procedure (Fig. 5B). Readmission rates (recorded in the databases since 2012) were lower following MI appendectomies (2.22 vs. 3.36%, *p* = 0.001) and cholecystectomies (3.19 vs. 6.25%, *p* ≤ 0.001). In contrast, slightly higher readmission rates for MI rectal resections were observed (6.18 vs. 4.58%, *p* = 0.017). No difference was found for right (5.67 vs 4.46%, *p* = 0.203) and left hemicolectomies (4.24 vs. 3.74%, *p* = 0.348) or gastrectomies (6.46 vs. 4.42%, *p* = 0.208). Adjusted in-hospital mortality rates were invariably lower after MIS cholecystectomies, while no relevant differences were observed for appendectomies and all 4 oncological procedures (Fig. 5C).

## Discussion

This nationwide study across 6 different surgical procedures revealed 5 consistent key factors decreasing the likelihood to receive MIS: elderly age, increased comorbidities, lack of private insurance, rural residence, and lower income. We found a strong interdependence among rural residence, low income, and decreased rate of private insurance coverage, suggesting that lower socio-economic status hindered MIS access.

Inequalities in access to modern surgical practice like MIS based on socioeconomic and demographic factors should not occur in an ideal healthcare system. However, multiple previous studies have shown higher rates of perioperative complications, decreased MIS amenability and, in case of oncological diseases, impaired long-term survival outcomes for members of certain ethnic groups [36, 37] and under-insured patients [25, 38, 39]. In contrast, the role of residential area and geographical location has just recently come into focus. Recent studies reported impaired access to laparoscopic surgery for diverticular disease and colorectal cancer [40–42] as well as modern surgical oncological care for patients living in rural areas in the US [43]. However, little is known how differences in residential areas within other countries influence surgical practice. Our findings of decreased MIS access of patients in rural areas in Switzerland are in accordance with previous reports, point towards a global phenomenon and probably reflect subpar training in oncologic MIS of less-specialized surgeons in remote areas.

The current report provides a comprehensive nationwide temporal analysis of the implementation of MIS over 20 years across different operations, ranging from simple procedures like appendectomy to complex oncological procedures like rectal resections or gastrectomies. As expected, rates of MIS increased across time for all procedures investigated. The data at hand pinpoint several interesting facts. Laparoscopic appendectomy was the first MIS visceral operation performed after the technique was adapted from gynecology [44]. However, laparoscopic cholecystectomy was the first MIS procedure to swiftly gain wide acceptance [45]. Indeed, by 1998, the start of our records, already three quarter of all cholecystectomies were performed laparoscopically. In contrast, implementation of laparoscopic appendectomy was slower, ranging below 25% in 1998 and reaching > 50% only by 2005. MIS implementation in more complex oncological procedures like hemicolectomy or rectal resections was considerably slower, although several large-scale randomized trials started reporting similar oncological short- and later long-term outcomes since 2002 [2, 7–9, 11, 45, 46]. Reasons for slow implementation likely include the learning curve for more complex procedures, which might be accentuated in the Swiss decentralized health care system. Switzerland has one of the highest numbers of physicians and hospitals per inhabitants [47], which results in limited caseloads of certain surgical conditions such as resections for colorectal cancer per surgeon and institution. Given that the learning curve for laparoscopic colorectal resections is 30–60 cases [48–50], the potential to learn MIS techniques adequately may be limited in rural, low volume hospitals. Furthermore, surgical training in Switzerland includes abdominal general surgery as well as musculoskeletal trauma and many general surgeons in smaller, peripheral Swiss hospitals still perform a broad spectrum of surgical procedures, preventing adequate specialization. Additionally, the implementation of working hour directives and the increased number of surgical residents have resulted in a decreased exposure to colorectal surgery during surgical training, which might furthermore impact on learning of complex MIS techniques [51]. However, centralization with resulting increased caseloads at specialized centers may result in increased MIS amenability for rural and low-income populations in the future. Further reasons might be owed to lack of specific instruments as well as a reluctance of older surgeons to learn new techniques [27], which necessitates the coming in charge of younger surgeons eager to promote modern techniques. Even so, rates observed in our analyses are not vastly different from countries with more pronounced specialization and higher hospital caseloads. In 2004, rates of MIS hemicolectomies for cancer were 4.3% [42], rising to 30–50% in 2009 [41, 52, 53] and 53.5% in 2012 [40] in the US. A recent report has shown US hospitals to be low MIS utilizers for cancer surgery [54] with rates similar to the percentages reported in our present study. Lastly, the lack of implementation of MIS gastrectomy is probably related to the small caseload in Europe, preventing an acceptable learning curve within a meaningful period [55].

We observed that overall degrees of comorbidities increased across all procedures studied, an effect probably dually owed to increased reporting of concomitant diseases in the databases and increasing patient age and comorbidities. Decreased MIS access of older and sicker patients is no novel finding and probably owed to multiple factors like e.g. hesitance to use pneumoperitoneum in patients with severe cardiopulmonary comorbidities or morbid obesity [56]. These results illustrate the paradox associated with MIS: Patients with advanced age and more comorbidities, who would likely benefit the most from the early postoperative advantages of MIS such as reduced pain, facilitated mobilization, faster bowel recovery and reduced hospital stay are more frequently denied MIS for reasons that remain elusive today. Positively, no discrimination in access to MIS based on gender or nationality was found; indeed, rates of MIS in women were higher in appendectomy and cholecystectomy. Furthermore, patients with foreign citizenship benefitted of increased rates of minimally invasive approaches in appendectomy, right hemicolectomies and rectal resections, a finding which might be related to the area of residence. Compared to Swiss citizens, a higher percentage of foreign individuals lives in urban (33.3 ± 1.28% foreign vs. 25.2 ± 1.08%, Swiss) and suburban (46.71 ± 1.94% foreign vs. 43.65 ± 1.18% Swiss) as opposed to rural residential areas (19.96 ± 1.36% foreign vs. 31.26 ± 1.64% Swiss) for all 6 procedures (all *p* ≤ 0.001). Residence in urban areas might have translated into improved access to specialized centers offering MIS access earlier and more frequently.

Finally, comparison of short-term outcomes using matched patients revealed striking differences in LOS related to the use of MIS. Population-wide benefits with respect to LOS appear larger than expected by results of RCTs, which normally report 1–2 days differences in LOS between open and MIS approaches in colectomy [5]. In our analysis, we found differences in LOS of up to 5 days for cholecystectomies and 3 days for colectomies, rectal resections and gastrectomies. With over 75% of cholecystectomies already being performed MI at the beginning of the study period, the striking difference in LOS of cholecystectomies is probably related to the higher level of comorbidities in patients undergoing open cholecystectomy, reserving OS for difficult cases. Furthermore, the larger than expected differences in LOS in colectomy may reflect disparities typically observed between RCT patient care maps and real-world data derived from observational studies. While some authors suggest that the benefits observed in clinical trials might be even more pronounced if biases are accounted for [57], multiple studies and statistical calculations show that results implied by small scale randomized or observations trials might fail to provide an accurate picture of nationwide developments [58]. Furthermore, LOS observed in our study were longer then reported from large-scale retrospective series from other countries. Switzerland has traditionally longer LOS than other countries in Europe or in the US [28], an aspect which is attributed to regional and cultural traditions rather than medical necessities. Lastly, the observed decrease in LOS across 20 years was modest at best despite major advances in surgical care, changes in hospital reward policies and advent of enhanced recovery after surgery programs [59]. Similarly, rates of readmissions, morbidities and mortality were higher than reported in selected populations of RCTs [5]. The strongly elevated rate of mortality after open cholecystectomy are probably owed to the extent of comorbidities in patients selected for open surgery. Mortality rates of colectomies were several years above 5%, similar to reports published around the change of the millennium [60], and have stagnated around 2% in the last years for left colectomy and rectal resection. Mortality rates after right hemicolectomy were elevated at 3.4%, similar to rates observed for open right hemicolectomy in a recent meta-analysis [61]. It is concerning, that no consistent morbidity and mortality benefits are observed for any colorectal MIS intervention on a national level. All these phenomena might depict a “real-life” effect, representing a median of all patients and not just well-selected patients for participation in a RCT [62].

The databases of all hospitalizations in Switzerland are primarily intended for administrative statistical evaluations by the responsible federal offices and the present study, therefore, has inherent limitations. As it is not a surgical quality control database, no specific information on the interventions itself (duration of surgery, experience of the operating surgeon, R- and N-status) are provided. Similarly, not intended as a cancer-registry, no conclusion on oncological outcomes can be drawn. Furthermore, the database in its current form does not provide information on the influence of center size, caseload, or conversion rates. While we provide absolute numbers for completely performed MIS procedures, we can only speculate how rates of conversion developed over time. However, other groups and we have reported conversion rates e.g. for colectomy [28, 40] and associated risk factors [63]. Lastly, national data of Switzerland might just be partly generalizable to other countries, especially with different healthcare systems. However, despite these limitations, the population-based nature of the databases and its large caseload provides a high level of generalizability and mirrors the actual adoption of MIS over 20 years.

## Conclusion

Strong socio-economic, geographic and demographic disparities exist in access to MIS techniques. Considering the observed benefits, efforts should be made to ensure equal MIS availability for patients with elderly age, increased comorbidities, lack of private insurance as well as residence in rural and low-income areas.

## Electronic supplementary material

Below is the link to the electronic supplementary material.

Electronic supplementary material 1 (DOCX 15 kb)

Electronic supplementary material 2 (DOCX 18 kb)

Electronic supplementary material 3 (DOCX 15 kb)

Electronic supplementary material 4 (DOCX 27 kb)

Electronic supplementary material 5 (DOCX 27 kb)

## Data Availability

The national databases covering all stationary hospitalizations is available from the BFS upon signature of a research & data protection agreement for a fee of 712 Swiss francs. All other data used in this study are freely available under the given links. All codes used for filtering, analyses and graphics are available from the first author upon reasonable request.

## Abbreviations

CI: 95% Confidence intervals
IQR: Interquartile range
LOS: Length of hospital stay
MIS: Minimally invasive surgery
OS: Open surgery
SD: Standard deviation
CRC: Colorectal cancer
ICD-10: International Classification of Diseases, German modification
BFS: Swiss federal statistical office
CHOP: Swiss surgical classification codes
MedStat regions: Place of residence with concomitant anonymity of single cases in 706 subdistrict geographical clusters
*n*: Number
%: Percentage
US: United States
