# Supplementary material for: Inequalities in access to minimally invasive general surgery: a comprehensive nationwide analysis across 20 years
Source: Surg Endosc. 2020 Nov 18;35(11):6227–43. doi: 10.1007/s00464-020-08123-0 (PMC8523463; doi:10.1007/s00464-020-08123-0)
Supplement: Supplementary file 1 — Electronic supplementary material 1 (DOCX 15 kb) [file 464_2020_8123_MOESM1_ESM.docx]

| **Supplementary Table 1: ICD-10 codes** **for filtering of patients’ diagnosis** | | |
| --- | --- | --- |
| **Disease** | **ICD-10 code** | **ICD-10 description** |
| **Appendicitis** | K35 | Acute appendicitis |
|  | K36 | Other appendicitis |
|  | K37 | Unspecified appendicitis |
| **Cholecystitis** | K80.0 | Calculus of gallbladder with acute cholecystitis |
|  | K80.1 | Calculus of gallbladder with other cholecystitis |
|  | K81.0 | Acute cholecystitis |
| **Right sided colorectal carcinoma** | C18.0 | Malignant neoplasm of colon - Caecum |
|  | C18.2 | Malignant neoplasm of colon - Ascending colon |
|  | C18.3 | Malignant neoplasm of colon - Hepatic flexure |
| **Left sided colorectal carcinoma** | C18.5 | Malignant neoplasm of colon - Splenic flexure |
|  | C18.6 | Malignant neoplasm of colon - Descending colon |
|  | C18.7 | Malignant neoplasm of colon - Sigmoid colon |
|  | C19 | Malignant neoplasm of rectosigmoid junction |
| **Rectal carcinoma** | C20 | Malignant neoplasm of rectum |
| **Gastric carcinoma** | C16 | Malignant neoplasm of stomach |
