# Supplementary material for: Inequalities in access to minimally invasive general surgery: a comprehensive nationwide analysis across 20 years
Source: Surg Endosc. 2020 Nov 18;35(11):6227–43. doi: 10.1007/s00464-020-08123-0 (PMC8523463; doi:10.1007/s00464-020-08123-0)
Supplement: Supplementary file 2 — Electronic supplementary material 2 (DOCX 18 kb) [file 464_2020_8123_MOESM2_ESM.docx]

| **Supplementary Table 2: Swiss surgical (CHOP) codes for filtering of patients’ procedures** | | |
| --- | --- | --- |
| **Intervention** | **Approach** | **Swiss surgical CHOP codes** |
| **Appendectomy** | Open | 470 ; 4700 ; 4702 ; 4709 |
|  | Laparoscopic | 4701  or any of the codes in section “open” in conjunction with 5421(laparoscopy as approach way) |
| **Cholecystectomy** | Open | 512 ; 5121 ; 5122 ; 512200 ; 512210 ; 512211 ; 512212 ; 512299 |
|  | Laparoscopic | 5123 ; 5124  or any of the codes in section “open” in conjunction with 5421(laparoscopy as approach way) |
|  | Robotic | Any of the codes in section “open” or “laparoscopic” in conjunction with 00995 (application of an operation robot) |
| **Right hemicolectomy** | Open | 457 ; 4571 ; 4573 ; 457311 ; 457711 ; 4579 ; 457900 ; 457910 ; 457921 ; 457923 ; 457924 ; 457925 ; 457926 ; 457929 ; 457999 ; 458111 |
|  | Laparoscopic | 457312 ; 457712 ; 458112  or any of the codes in section “open” in conjunction with 5421(laparoscopy as approach way) |
|  | Robotic | Any of the codes in section “open” or “laparoscopic” in conjunction with 00995 (application of an operation robot) |
| **Left hemicolectomy** | Open | 457 ; 4570 ; 4571 ; 4575 ; 457511 ; 457521 ; 4576 ; 457611 ; 457721 ; 4579 ; 457900 ; 457910 ; 457912 ; 457920 ; 457921 ; 457922 ; 457923 ; 457924 ; 457925 ; 457926 ; 457929 ; 457999 ; 458 ; 458111 ; 4589 ; 458X00 ; 458X20 ; 458X30 ; 458X99 ; 484 ; 4841 ; 4849 ; 485 ; 4851 ; 485X00 ; 485X10 ; 485X11 ; 485X99 ; 486 ; 4861 ; 4862 ; 4863 ; 486300 ; 486310 ; 486399 ; 4864 ; 486499 ; 4865 ; 486609 ; 486612 ; 486621 ; 486622 ; 486631 ; 486632 ; 486635 ; 4869 ; 486900 ; 486910 ; 486999 |
|  | Laparoscopic | 457512 ; 457522 ; 457621 ; 457722 ; 486410 ; 486611 ; 486614 ; 486623 ; 486624 ; 486633 ; 486634 ; 486911  or any of the codes in section “open” in conjunction with 5421(laparoscopy as approach way) |
|  | Robotic | Any of the codes in section “open” or “laparoscopic” in conjunction with 00995 (application of an operation robot) |
| **Rectal resection** | Open | 484 ; 4841 ; 4849 ; 485 ; 4851 ; 4852 ; 4853 ; 4854 ; 4859 ; 485X00 ; 485X10 ; 485X11 ; 485X99 ; 486 ; 4861 ; 4862 ; 4863 ; 486300 ; 486310 ; 486399 ; 4864 ; 486410 ; 486499 ; 4865 ; 486609 ; 486615 ; 486621 ; 486622 ; 486631 ; 486632 ; 486635 ; 4869 ; 486900 ; 486910 ; 486911 ; 486999 |
|  | Laparoscopic | 486623 ; 486624 ; 486625 ; 486633 ; 486634  or any of the codes in section “open” in conjunction with 5421(laparoscopy as approach way) |
|  | Robotic | Any of the codes in section “open” or “laparoscopic” in conjunction with 00995 (application of an operation robot) |
| **Gastrectomy** | Open | 435 ; 436 ; 436X00 ; 436X10 ; 436X99 ; 437 ; 437111 ; 437121 ; 437211 ; 437221 ; 4379 ; 437X00 ; 437X10 ; 437X20 ; 437X99 ; 438 ; 4381 ; 4389 ; 438900 ; 438909 ; 438910 ; 438961 ; 438990 ; 438999 ; 439 ; 4391 ; 4392 ; 4399 ; 439900 ; 439999 |
|  | Laparoscopic | Any of the codes in section “open” in conjunction with 5421(laparoscopy as approach way) |
|  | Robotic | Any of the codes in section “open” or “laparoscopic” in conjunction with 00995 (application of an operation robot) |
