# Supplementary material for: Inequalities in access to minimally invasive general surgery: a comprehensive nationwide analysis across 20 years
Source: Surg Endosc. 2020 Nov 18;35(11):6227–43. doi: 10.1007/s00464-020-08123-0 (PMC8523463; doi:10.1007/s00464-020-08123-0)
Supplement: Supplementary file 3 — Electronic supplementary material 3 (DOCX 15 kb) [file 464_2020_8123_MOESM3_ESM.docx]

| **Supplementary Table 3: ICD-10 codes for assessment of complications** | | |
| --- | --- | --- |
| **Disease** | **ICD-10 code** | **ICD-10 description** |
| **Wound infection** | T81.4 | Infection following a procedure, not elsewhere classified, including Abscess (intra-abdominal, stitch, subphrenic, wound) & Sepsis |
|  | K65.0 | Acute peritonitis, including Abscess (abdominopelvic, mesenteric, omentum, peritoneum, retrocaecal, retroperitoneal, subdiaphragmatic, subhepatic, subphrenic) |
|  | K65.9 | Peritonitis, unspecified |
| **Bleeding & hematoma** | T81.0 | Haemorrhage and haematoma complicating a procedure, not elsewhere classified |
| **Anastomotic insufficiency /**  **Gastrointestinal leakage** | K91.83 | Insufficiency of anastomosis or stitches after operations at the gastrointestinal tract |
