# Supplementary material for: Inequalities in access to minimally invasive general surgery: a comprehensive nationwide analysis across 20 years
Source: Surg Endosc. 2020 Nov 18;35(11):6227–43. doi: 10.1007/s00464-020-08123-0 (PMC8523463; doi:10.1007/s00464-020-08123-0)
Supplement: Supplementary file 4 — Electronic supplementary material 4 (DOCX 27 kb) [file 464_2020_8123_MOESM4_ESM.docx]

| **Supplementary Table 4: Patient demographic data after propensity score matching** | | | | | | | | | | | | | | | | | | | | | | | | | | | | | | | | | | | | |  |
| --- | --- | --- | --- | --- | --- | --- | --- | --- | --- | --- | --- | --- | --- | --- | --- | --- | --- | --- | --- | --- | --- | --- | --- | --- | --- | --- | --- | --- | --- | --- | --- | --- | --- | --- | --- | --- | --- |
|  | **Appendectomy**  **(n=72878)** | | | | | | **Cholecystectomy**  **(n=12420)** | | | | | | **Right Hemicolectomy**  **(n=3517)** | | | | | | **Left Hemicolectomy**  **(n=11530)** | | | | | | **Rectal Resection**  **(n=7220)** | | | | | | **Gastrectomy**  **(n=1757)** | | | | | |  |
|  | **OS** | | **MIS** | | **P-value** | | **OS** | | **MIS** | | **P-value** | | **OS** | | **MIS** | | **P-value** | | **OS** | | **MIS** | | **P-value** | | **OS** | | **MIS** | | **P-value** | | **OS** | | **MIS** | | **P-value** | |  |
|  | **(n=36439)** | | **(n=36439)** | |  |  | **(n=6210)** | | **(n=6210)** | |  |  | **(n=1877)** | | **(n=1640)** | |  |  | **(n=6251)** | | **(n=5279)** | |  |  | **(n=3859)** | | **(n=3361)** | |  |  | **(n=1273)** | | **(n=484)** | |  |  |  |
| **Gender** |  | |  | |  | |  | |  | |  | |  | |  | |  | |  | |  | |  | |  | |  | |  | |  | |  | |  | |  |
| Male | 20800 (57.1%) | | 20190 (55.4%) | | <0.001 | | 3534 (56.9%) | | 3499 (56.3%) | | 0.538 | | 946 (50.4%) | | 830 (50.6%) | | 0.919 | | 3693 (59.1%) | | 3099 (58.7%) | | 0.698 | | 2360 (61.2%) | | 2077 (61.8%) | | 0.577 | | 772 (60.6%) | | 280 (57.9%) | | 0.301 | |  |
| Female | 15639 (42.9%) | | 16249 (44.6%) | |  | | 2676 (43.1%) | | 2711 (43.7%) | |  | | 931 (49.6%) | | 810 (49.4%) | |  | | 2558 (40.9%) | | 2180 (41.3%) | |  | | 1499 (38.8%) | | 1284 (38.2%) | |  | | 501 (39.4%) | | 204 (42.1%) | |  | |  |
| **Age (Years)** |  | |  | |  | |  | |  | |  | |  | |  | |  | |  | |  | |  | |  | |  | |  | |  | |  | |  | |  |
| 00-19 | 13337 (36.6%) | | 12114 (33.2%) | | <0.001 | | 13 (0.2%) | | 12 (0.2%) | | <0.001 | | 0 (0%) | | 0 (0%) | | 0.848 | | 1 (0.0%) | | 0 (0%) | | 0.796 | | 0 (0%) | | 0 (0%) | | 0.349 | | 0 (0%) | | 0 (0%) | | 0.598 | |  |
| 20-39 | 11376 (31.2%) | | 11483 (31.5%) | |  | | 264 (4.3%) | | 443 (7.1%) | |  | | 10 (0.5%) | | 12 (0.7%) | |  | | 83 (1.3%) | | 76 (1.4%) | |  | | 66 (1.7%) | | 66 (2.0%) | |  | | 36 (2.8%) | | 12 (2.5%) | |  | |  |
| 40-59 | 7522 (20.6%) | | 8317 (22.8%) | |  | | 1200 (19.3%) | | 1213 (19.5%) | |  | | 244 (13.0%) | | 222 (13.5%) | |  | | 1337 (21.4%) | | 1165 (22.1%) | |  | | 913 (23.7%) | | 848 (25.2%) | |  | | 297 (23.3%) | | 114 (23.6%) | |  | |  |
| 60-79 | 3499 (9.6%) | | 3794 (10.4%) | |  | | 3179 (51.2%) | | 3083 (49.6%) | |  | | 1077 (57.4%) | | 931 (56.8%) | |  | | 3751 (60.0%) | | 3146 (59.6%) | |  | | 2297 (59.5%) | | 1948 (58.0%) | |  | | 773 (60.7%) | | 283 (58.5%) | |  | |  |
| 80-99 | 705 (1.9%) | | 731 (2.0%) | |  | | 1554 (25.0%) | | 1459 (23.5%) | |  | | 546 (29.1%) | | 475 (29.0%) | |  | | 1079 (17.3%) | | 892 (16.9%) | |  | | 583 (15.1%) | | 499 (14.8%) | |  | | 167 (13.1%) | | 75 (15.5%) | |  | |  |
| **Nationality** |  | |  | |  | |  | |  | |  | |  | |  | |  | |  | |  | |  | |  | |  | |  | |  | |  | |  | |  |
| Swiss | 30106 (82.6%) | | 29762 (81.7%) | | 0.001 | | 5467 (88.0%) | | 5401 (87.0%) | | 0.078 | | 1635 (87.1%) | | 1425 (86.9%) | | 0.888 | | 5418 (86.7%) | | 4527 (85.8%) | | 0.158 | | 3324 (86.1%) | | 2868 (85.3%) | | 0.345 | | 1002 (78.7%) | | 384 (79.3%) | | 0.794 | |  |
| Foreign | 6333 (17.4%) | | 6677 (18.3%) | |  | | 743 (12.0%) | | 809 (13.0%) | |  | | 242 (12.9%) | | 215 (13.1%) | |  | | 833 (13.3%) | | 752 (14.2%) | |  | | 535 (13.9%) | | 493 (14.7%) | |  | | 271 (21.3%) | | 100 (20.7%) | |  | |  |
| **Area of Residence** |  | |  | |  | |  | |  | |  | |  | |  | |  | |  | |  | |  | |  | |  | |  | |  | |  | |  | |  |
| Urban City | 8966 (24.6%) | | 8757 (24.0%) | | 0.027 | | 1490 (24.0%) | | 1461 (23.5%) | | 0.052 | | 541 (28.8%) | | 458 (27.9%) | | 0.744 | | 1751 (28.0%) | | 1468 (27.8%) | | 0.407 | | 1082 (28.0%) | | 919 (27.3%) | | 0.641 | | 420 (33.0%) | | 159 (32.9%) | | 0.969 | |  |
| Smaller Towns & Suburbs | 15227 (41.8%) | | 15577 (42.7%) | |  | | 2555 (41.1%) | | 2685 (43.2%) | |  | | 869 (46.3%) | | 780 (47.6%) | |  | | 2974 (47.6%) | | 2466 (46.7%) | |  | | 1732 (44.9%) | | 1501 (44.7%) | |  | | 505 (39.7%) | | 195 (40.3%) | |  | |  |
| Rural Area | 12246 (33.6%) | | 12105 (33.2%) | |  | | 2165 (34.9%) | | 2064 (33.2%) | |  | | 467 (24.9%) | | 402 (24.5%) | |  | | 1526 (24.4%) | | 1345 (25.5%) | |  | | 1045 (27.1%) | | 941 (28.0%) | |  | | 348 (27.3%) | | 130 (26.9%) | |  | |  |
| **Taxable Income per Region** | | | |  | |  | |  | |  | |  | |  | |  | |  | |  | |  | |  | |  | |  | |  | |  | |  | |  | |
| 0-19 Percentile | | 8551 (23.5%) | | 6751 (18.5%) | | <0.001 | | 1433 (23.1%) | | 1409 (22.7%) | | <0.001 | | 346 (18.4%) | | 291 (17.7%) | | 0.001 | | 1244 (19.9%) | | 916 (17.4%) | | <0.001 | | 740 (19.2%) | | 623 (18.5%) | | <0.001 | | 221 (17.4%) | | 75 (15.5%) | | 0.416 | |
| 20-39 Percentile | | 7866 (21.6%) | | 7134 (19.6%) | |  | | 1371 (22.1%) | | 1170 (18.8%) | |  | | 399 (21.3%) | | 273 (16.6%) | |  | | 1220 (19.5%) | | 977 (18.5%) | |  | | 793 (20.5%) | | 641 (19.1%) | |  | | 253 (19.9%) | | 85 (17.6%) | |  | |
| 40-59 Percentile | | 6779 (18.6%) | | 7789 (21.4%) | |  | | 1173 (18.9%) | | 1235 (19.9%) | |  | | 349 (18.6%) | | 323 (19.7%) | |  | | 1190 (19.0%) | | 1031 (19.5%) | |  | | 781 (20.2%) | | 615 (18.3%) | |  | | 261 (20.5%) | | 117 (24.2%) | |  | |
| 60-79 Percentile | | 6672 (18.3%) | | 7028 (19.3%) | |  | | 1072 (17.3%) | | 1231 (19.8%) | |  | | 335 (17.8%) | | 366 (22.3%) | |  | | 1118 (17.9%) | | 1163 (22.0%) | |  | | 772 (20.0%) | | 849 (25.3%) | |  | | 278 (21.8%) | | 109 (22.5%) | |  | |
| 80-100 Percentile | | 6571 (18.0%) | | 7737 (21.2%) | |  | | 1161 (18.7%) | | 1165 (18.8%) | |  | | 448 (23.9%) | | 387 (23.6%) | |  | | 1479 (23.7%) | | 1192 (22.6%) | |  | | 773 (20.0%) | | 633 (18.8%) | |  | | 260 (20.4%) | | 98 (20.2%) | |  | |
