# Supplementary material for: Inequalities in access to minimally invasive general surgery: a comprehensive nationwide analysis across 20 years
Source: Surg Endosc. 2020 Nov 18;35(11):6227–43. doi: 10.1007/s00464-020-08123-0 (PMC8523463; doi:10.1007/s00464-020-08123-0)
Supplement: Supplementary file 5 — Electronic supplementary material 5 (DOCX 27 kb) [file 464_2020_8123_MOESM5_ESM.docx]

| **Supplementary Table 5: Baseline surgical and hospitalisation data after propensity score matching** | | | | | | | | | | | | | | | | | | | | | | | | | | | | | | | | | | | | |
| --- | --- | --- | --- | --- | --- | --- | --- | --- | --- | --- | --- | --- | --- | --- | --- | --- | --- | --- | --- | --- | --- | --- | --- | --- | --- | --- | --- | --- | --- | --- | --- | --- | --- | --- | --- | --- |
|  | **Appendectomy**  **(n=72878)** | | | | | | **Cholecystectomy**  **(n=12420)** | | | | | | **Right Hemicolectomy**  **(n=3517)** | | | | | | | **Left Hemicolectomy**  **(n=11530)** | | | | | | **Rectal Resection**  **(n=7220)** | | | | | | **Gastrectomy**  **(n=1757)** | | | | |
|  | **OS** | | **MIS** | | **P-value** | | **OS** | | **MIS** | | **P-value** | | **OS** | | **MIS** | | **P-value** | | | **OS** | | **MIS** | | **P-value** | | **OS** | | **MIS** | | **P-value** | | **OS** | | **MIS** | | **P-value** |
|  | **(n=36439)** | | **(n=36439)** | |  |  | **(n=6210)** | | **(n=6210)** | |  |  | **(n=1877)** | | **(n=1640)** | |  |  |  | **(n=6251)** | | **(n=5279)** | |  |  | **(n=3859)** | | **(n=3361)** | |  |  | **(n=1273)** | | **(n=484)** | |  |
| **Surgical Technique** |  | |  | |  | |  | |  | |  | |  | |  | |  | | |  | |  | |  | |  | |  | |  | |  | |  | |  |
| Open Surgery | 36439 (100%) | | 0 (0%) | | <0.001 | | 6210 (100%) | | 0 (0%) | | <0.001 | | 1877 (100%) | | 0 (0%) | | <0.001 | | | 6251 (100%) | | 0 (0%) | | <0.001 | | 3859 (100%) | | 0 (0%) | | <0.001 | | 1273 (100%) | | 0 (0%) | | <0.001 |
| Laparoscopic Surgery | 0 (0%) | | 36439 (100%) | |  | | 0 (0%) | | 6207 (100.0%) | |  | | 0 (0%) | | 1615 (98.5%) | |  | | | 0 (0%) | | 5229 (99.1%) | |  | | 0 (0%) | | 3194 (95.0%) | |  | | 0 (0%) | | 451 (93.2%) | |  |
| Robotic Surgery | 0 (0%) | | 0 (0%) | |  | | 0 (0%) | | 3 (0.0%) | |  | | 0 (0%) | | 25 (1.5%) | |  | | | 0 (0%) | | 50 (0.9%) | |  | | 0 (0%) | | 167 (5.0%) | |  | | 0 (0%) | | 33 (6.8%) | |  |
| **Year of Operation** |  | |  | |  | |  | |  | |  | |  | |  | |  | | |  | |  | |  | |  | |  | |  | |  | |  | |  |
| 1998-2002 | 8502 (23.3%) | | 8502 (23.3%) | | 1 | | 1634 (26.3%) | | 1634 (26.3%) | | 1 | | 61 (3.2%) | | 31 (1.9%) | | <0.001 | | | 469 (7.5%) | | 236 (4.5%) | | <0.001 | | 206 (5.3%) | | 105 (3.1%) | | <0.001 | | 152 (11.9%) | | 31 (6.4%) | | <0.001 |
| 2003-2007 | 15167 (41.6%) | | 15167 (41.6%) | |  | | 1626 (26.2%) | | 1626 (26.2%) | |  | | 417 (22.2%) | | 210 (12.8%) | |  | | | 1493 (23.9%) | | 754 (14.3%) | |  | | 795 (20.6%) | | 398 (11.8%) | |  | | 381 (29.9%) | | 80 (16.5%) | |  |
| 2008-2012 | 9806 (26.9%) | | 9806 (26.9%) | |  | | 1502 (24.2%) | | 1502 (24.2%) | |  | | 515 (27.4%) | | 515 (31.4%) | |  | | | 2021 (32.3%) | | 2021 (38.3%) | |  | | 1148 (29.7%) | | 1148 (34.2%) | |  | | 312 (24.5%) | | 156 (32.2%) | |  |
| 2013-2017 | 2964 (8.1%) | | 2964 (8.1%) | |  | | 1448 (23.3%) | | 1448 (23.3%) | |  | | 884 (47.1%) | | 884 (53.9%) | |  | | | 2268 (36.3%) | | 2268 (43.0%) | |  | | 1710 (44.3%) | | 1710 (50.9%) | |  | | 428 (33.6%) | | 217 (44.8%) | |  |
| **Insurance Status** |  | |  | |  | |  | |  | |  | |  | |  | |  | | |  | |  | |  | |  | |  | |  | |  | |  | |  |
| Statutory | 30399 (83.4%) | | 29557 (81.1%) | | <0.001 | | 4663 (75.1%) | | 4674 (75.3%) | | 0.835 | | 1163 (62.0%) | | 1034 (63.0%) | | 0.507 | | | 3988 (63.8%) | | 3445 (65.3%) | | 0.105 | | 2522 (65.4%) | | 2262 (67.3%) | | 0.085 | | 892 (70.1%) | | 339 (70.0%) | | 1 |
| Private | 6040 (16.6%) | | 6882 (18.9%) | |  | | 1547 (24.9%) | | 1536 (24.7%) | |  | | 714 (38.0%) | | 606 (37.0%) | |  | | | 2263 (36.2%) | | 1834 (34.7%) | |  | | 1337 (34.6%) | | 1099 (32.7%) | |  | | 381 (29.9%) | | 145 (30.0%) | |  |
| **Length of Hospital Stay (Days)** | | | |  | |  | |  | |  | |  | |  | |  | |  |  | |  | |  | |  | |  | |  | |  | |  | |  | |
| Mean (SD) | | 4.66 (4.36) | | 4.13 (3.78) | | <0.001 | | 12.3 (9.51) | | 7.73 (11.8) | | <0.001 | | 14.8 (10.0) | | 14.3 (12.2) | | 0.211 | 15.0 (9.60) | | 13.3 (11.6) | | <0.001 | | 17.5 (12.7) | | 16.5 (13.7) | | 0.002 | | 20.5 (16.0) | | 18.7 (19.1) | | 0.063 | |
| Median [Q1, Q3] | | 3.00 [2.00, 5.00] | | 3.00 [2.00, 5.00] | | <0.001 | | 10.0 [7.00, 15.0] | | 6.00 [4.00, 9.00] | | <0.001 | | 12.0 [9.00, 18.0] | | 10.0 [7.00, 17.0] | | <0.001 | 12.0 [9.00, 18.0] | | 9.00 [7.00, 15.0] | | <0.001 | | 14.0 [11.0, 21.0] | | 12.0 [9.00, 19.0] | | <0.001 | | 16.0 [12.0, 24.0] | | 14.0 [9.00, 21.0] | | <0.001 | |
| **Comorbidities (Elixhauser Score)** | | | |  | |  | |  | |  | |  | |  | |  | |  |  | |  | |  | |  | |  | |  | |  | |  | |  | |
| Mean (SD) | | 0.286 (2.47) | | 0.293 (2.62) | | 0.714 | | 2.70 (6.43) | | 2.49 (6.08) | | 0.068 | | 12.5 (7.80) | | 12.8 (8.39) | | 0.259 | 11.7 (7.26) | | 11.9 (7.46) | | 0.097 | | 12.2 (7.50) | | 12.5 (7.75) | | 0.099 | | 11.7 (7.27) | | 12.7 (8.37) | | 0.035 | |
| Median [Q1, Q3] | | 0.00 [0.00, 0.00] | | 0.00 [0.00, 0.00] | | 0.147 | | 0.00 [0.00, 5.00] | | 0.00 [0.00, 4.00] | | 0.354 | | 11.0 [7.00, 16.0] | | 11.0 [7.00, 16.0] | | 0.924 | 8.00 [7.00, 14.0] | | 9.00 [7.00, 14.0] | | 0.120 | | 9.00 [7.00, 15.0] | | 10.0 [7.00, 16.0] | | 0.119 | | 9.00 [7.00, 14.0] | | 10.0 [7.00, 17.0] | | 0.242 | |
| **In-Hospital Mortality** | |  | |  | |  | |  | |  | |  | |  | |  | |  |  | |  | |  | |  | |  | |  | |  | |  | |  | |
| No | | 36382 (99.8%) | | 36404 (99.9%) | | 0.028 | | 6076 (97.8%) | | 6165 (99.3%) | | <0.001 | | 1813 (96.6%) | | 1585 (96.6%) | | 1 | 6084 (97.3%) | | 5160 (97.7%) | | 0.167 | | 3783 (98.0%) | | 3297 (98.1%) | | 0.864 | | 1222 (96.0%) | | 473 (97.7%) | | 0.083 | |
| Yes | | 57 (0.2%) | | 35 (0.1%) | |  | | 134 (2.2%) | | 45 (0.7%) | |  | | 64 (3.4%) | | 55 (3.4%) | |  | 167 (2.7%) | | 119 (2.3%) | |  | | 76 (2.0%) | | 64 (1.9%) | |  | | 51 (4.0%) | | 11 (2.3%) | |  | |
